# Transforming Rheumatoid Arthritis Care: A Scoping Review of the Role of Digital Health

**DOI:** 10.7759/cureus.93973

**Published:** 2025-10-06

**Authors:** Robin Sia, Ee Lynn Ting, Alexander Kwan, Mueed Mian

**Affiliations:** 1 Internal Medicine, Rheumatology, Northern Hospital, Epping, AUS; 2 Rheumatology, Northern Hospital, Epping, AUS; 3 Rheumatology, St Vincent's Hospital, Fitzroy, AUS

**Keywords:** digital app, digital application, digital health, digital health technology, mobile health technology, patient engagement, rheumatoid arthritis, rheumatology

## Abstract

This review explored the role of digital health in managing rheumatoid arthritis (RA), assessing interventions such as telemedicine, mobile apps, artificial intelligence, and electronic health records. A search of PubMed and the Cochrane Library yielded 976 articles, of which 10 met the inclusion criteria (six randomized controlled trials (RCTs) and four systematic reviews). Half of the RCTs showed improved outcomes, while the others found no significant benefit, and the systematic reviews highlighted substantial heterogeneity across interventions. Overall, digital health shows promise in RA care, but the mixed evidence underscores the need for larger, methodologically robust studies to clarify its effectiveness and guide integration into clinical practice.

## Introduction and background

Rheumatoid arthritis (RA) is a multi-etiological, autoimmune, and chronic inflammatory disorder often associated with pain, swelling, and morning stiffness with a symmetrical distribution of multiple peripheral joints, including metacarpophalangeal (MCPJ), metatarsophalangeal (MTPJ), and proximal interphalangeal joints (PIPJ) with other extra-articular manifestations, including serositis, vasculitis, Felty’s syndrome, pulmonary involvement, and peripheral neuropathy in the later course of the disease [1-3]. The worldwide prevalence of RA ranges from 0.4% to 1.3% [1]. Due to the fluctuating nature of RA, frequent symptom assessment is essential for an accurate representation of the disease [4,5]. There is a need for patient participation in both treatment decision-making and disease activity assessment. RA symptoms are traditionally assessed using patient-reported outcome (PRO) measures, such as patient global assessment (PGA), health assessment questionnaire (HAQ), visual analog scale (VAS), and duration of morning stiffness [6]. PRO measures, despite capturing useful information regarding the impact of their disease, are subjective and provide only a brief overview of their symptoms, especially when administered inconsistently [7]. To ensure a more accurate picture of the patient’s disease status, it is important to create novel approaches that have more frequent and sensitive monitoring of the patient’s disease impact and quality of life [8].

Digital technologies may provide an opportunity to not only continuously track and monitor a patient’s experience with RA but also provide a collection of patient-centric measurements of disease activity. Digital health is a broad term encompassing electronically captured data, as well as technical and communications infrastructure and applications in the healthcare ecosystem [9]. Over the past several decades, digital technology has rapidly advanced, and this has significantly impacted all aspects of human endeavour. The use of digital recording of physical status and experiences has set the stage for revolutionary progress in individual health, as well as medical management. Furthermore, the COVID-19 pandemic has drastically changed the management of rheumatic diseases with the increased risk of infection and the prolonging of appointments and monitoring intervals [10,11]. The use of digital technologies is not uncommon in rheumatic diseases only. They have also been utilized in neurodegenerative diseases, such as Parkinson’s disease and amyotrophic lateral sclerosis [12-14]. With this, there has been an increase in the utilization of digital health in clinical practice.

In this scoping review, we explore the use of various digital applications in the management of RA.

## Review

Methods

A comprehensive search of the literature was conducted in PubMed and the Cochrane Library for the period 2018-2023. Keywords included 'rheumatoid*' and 'digital*', separated by ‘AND’ and ‘OR’ Boolean operators. The PubMed search yielded 66 records, of which 11 articles met the predefined eligibility criteria. A parallel search in the Cochrane Library identified 96 records, none of which met the inclusion criteria.

**Figure 1:**
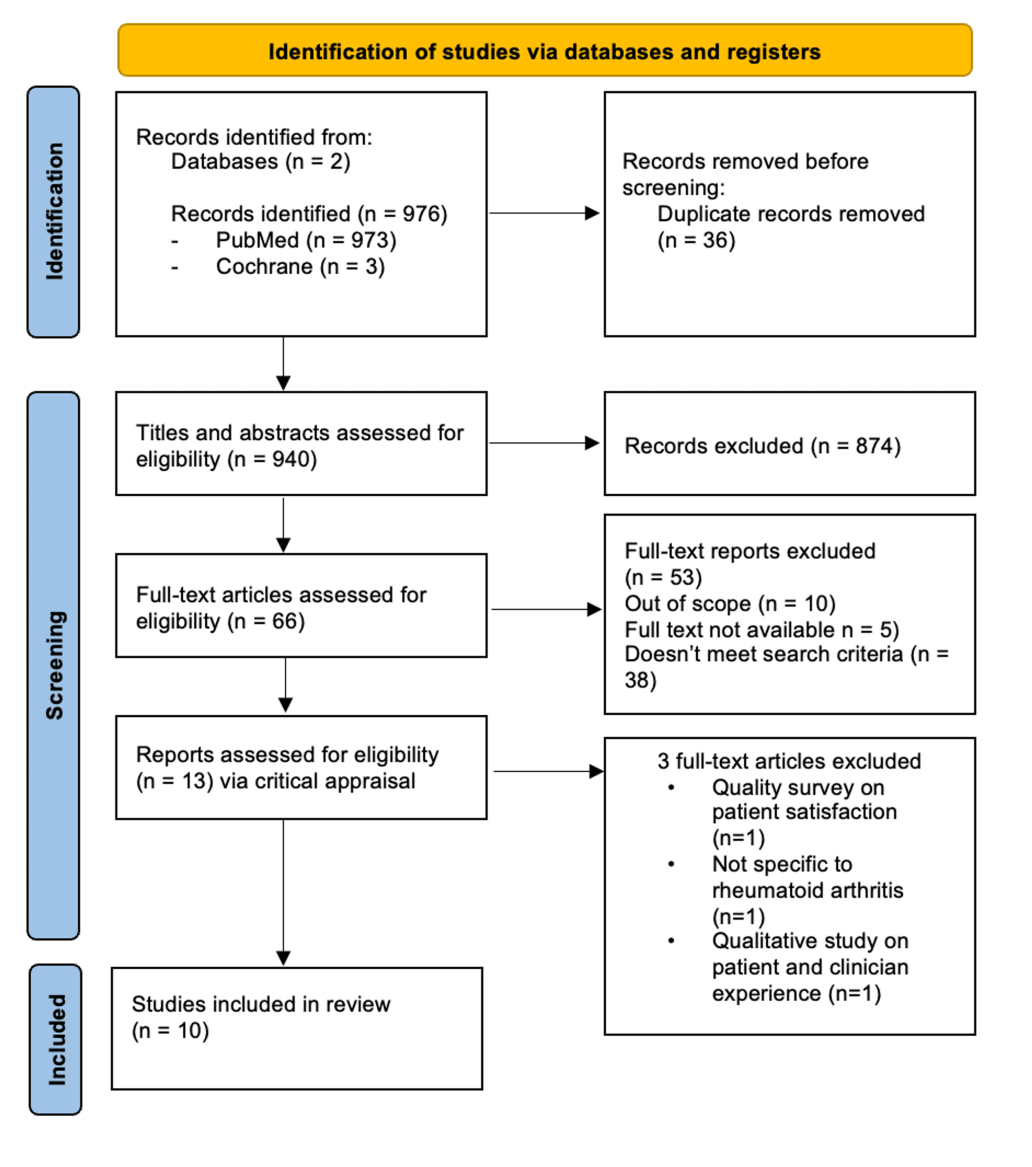
PRISMA flow diagram of the search, screening, and number of results obtained. PRISMA: Preferred Reporting Items for Systematic Reviews and Meta-Analyses

In total, the literature search retrieved 976 records. After removal of duplicates and application of inclusion criteria (English language, randomized controlled trials (RCTs), and systematic reviews focusing on digital health and apps in RA), 10 articles were selected for inclusion in this scoping review (Figure 1).

Results

**Figure 2:**
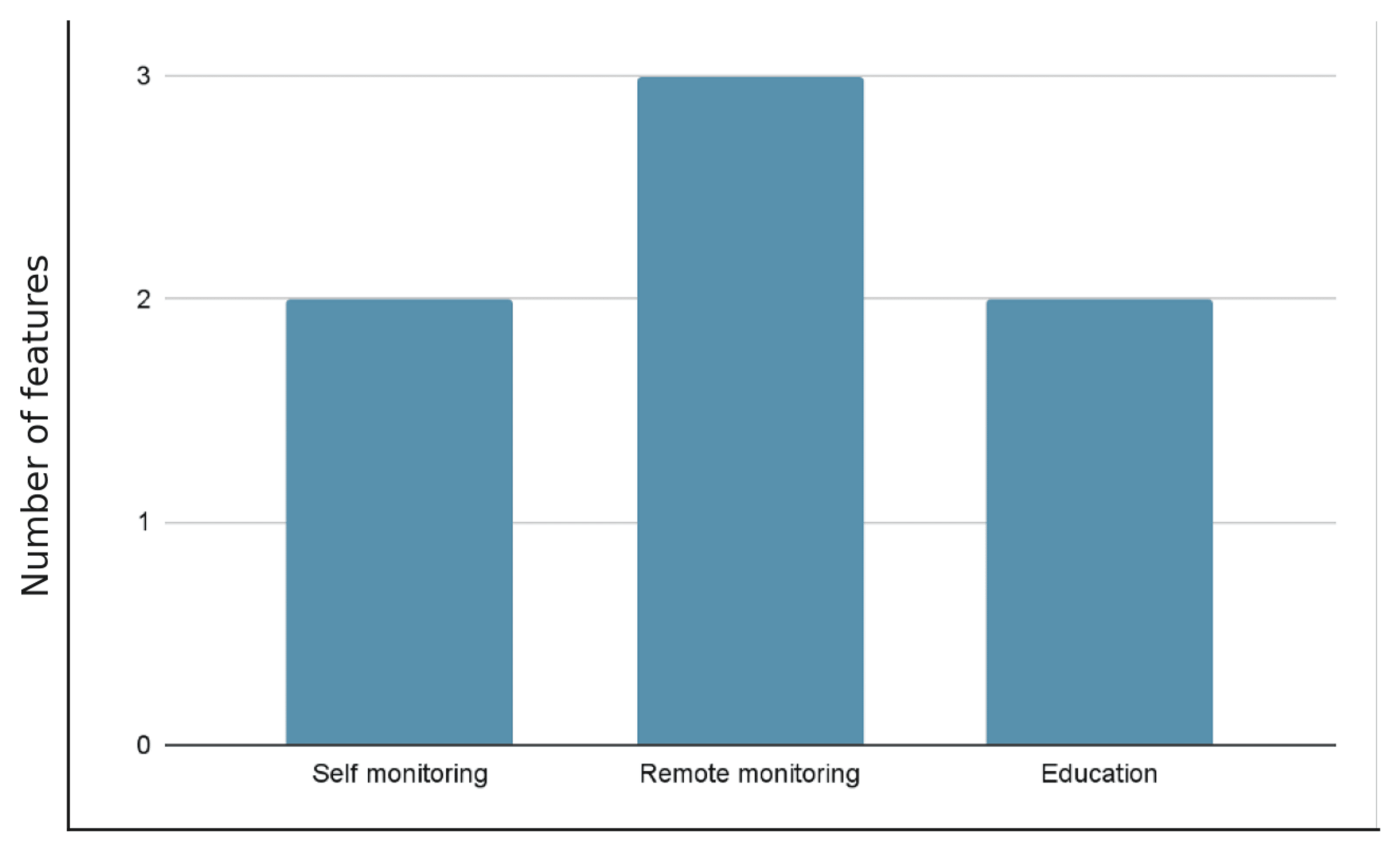
Number of features among the six RCT digital applicationss included in this review.

There were six RCTs and four systematic reviews identified. The study design varied among the studies. Table 1 provides a summary of the studies we have included in our review, demonstrating the heterogeneity of different articles and their results. Figure 2 demonstrates the different types of digital apps used in RA.

**Table 1:** Characteristics and results of the selected studies comparing intervention with usual care and their outcomes. Randomized Controlled Trial (RCT); Smart System of Disease Management Group (SSDM); The Disease Activity Score 28 (DAS28-CRP); High Intensity Interval Training (HIIT); Hand Function: Michigan Hand Outcome Questionnaire (MHQ); Analysis of Variance (ANOVA); Clinical Disease Activity Index (CDAI); Treatment Satisfaction Questionnaire for Medication (TSQM); Perceived Efficacy in Patient-Physician Interaction (PEPPI); Electronic Patient Reported Outcomes (ePRO); Hand Function: Michigan Hand Outcome Questionnaire (MHQ); Mobile App Rating Scale (MARS)

Study	Intervention	No. of patients	Primary Outcome	Results
Randomized Controlled Trials				
Li et al. (2023), China [15]	Patients randomised to use the Smart System for Disease Management (SSDM app)	2204	DAS28-CRP	DAS28-CRP of 3.2 or less was 71.0% (780 of 1099 patients) in the SSDM group vs 64.5% (708 of 1098 patients) in the control group (difference between groups, 6.6%; 95% CI, 2.7% to 10.4%; P=0.001).
Håvard Haglo et al. (2021) [16]	Smartphone app (Myworkout GO) - 4x4min HIIT guided by App Group (AG)	40	Measurement of VO2 max	Similar effects on VO2max (p<0.001) as conventional, supervised training carried out in a rehabilitation clinic.
Sánchez-Laulhé et al. (2022) [17]	Digital app (CareHand) - home exercise program, educational and self-management	30	MHQ	Better results in the short and medium term for overall hand function (P<.05 work performance pain and satisfaction with mean differences between groups for the total score of points ci using anova in mhq.)
Colls et al. (2020) USA [18]	Electronic patient reported outcomes (ePRO) app and adherence to the app	78	Clinical Disease Activity Index (CDAI)	Adherence to the app was higher among older age group (P=0.03) & better disease control (P=0.02).
Seppen et al. (2022) [19]	RAPID3 Smartphone app - Self-reporting with early identification of RA flare	103	DAS28-ESR	No significant difference in mean change in DAS28-ESR in both groups (0.27 vs 0.35 in usual care group) (95% CI −0.39, 0.30).
Lee et al. (2021), USA [20]	ePRO app	191	Patient satisfaction with treatment: -TSQM -PEPPI -CDAI	No group differences in medians of 6-month TSQM or PEPPI. No group differences in 6-month median CDAI (B -2.0, 95% CI -5.8, 1.8) were identified.
Systematic Reviews				
Dantas et al. (2021) Brazil [21]	Explored mHealth apps available in Brazil and their respect to engagement, interface, experience and information quality	5 out of 3173 apps	Used MARS scale to assess quality of the apps	Lack of quality mHealth apps, with poor aesthetics and interface. Low quality evidence-based information.
Luo et al. (2019) [22]	Systematic Review comparing 20 different apps intended for use for RA	20 out of 346 apps	4 Features assessed - basic characteristics, content source, functionality, security - each app was assessed individually (unable to compare)	Most current apps are not comprehensive and heterogeneous in what they offer (50% symptom tracking, 20% information about RA, 20% offered engagement, 50% provided ability to contact healthcare, 30% provided security.
Bearne et al. (2020), United Kingdom [23]	Assessing mobile apps on Apple App store and Google Play Store in UK	4 apps	App quality: Mobile App Rating Scale (MARS; 0-5)	Variable app quality with MARS score 2.25-4.17.
Seppen et al. (2020), Netherlands [24]	Systematic Review conducted in a Dutch setting looking into 10 RCTs (of 4 different asynchronous mHealth apps)	4 apps in 10 RCTs	4 Different types of asynchronous mHealth (SMS reminders, web apps, smartphone apps, pedometers)	Achieve remission sooner with asynchronous mHealth app via medication compliance and be more physically active via reminders.

Features of Digital Health Applications

Education and self- and remote monitoring: Currently, rheumatologists assess disease activity at discrete time points during clinic appointments. In contrast, ePRO may improve continuous monitoring of disease activity, which helps clinicians better understand the trajectory of a patient’s disease course. An RCT by Lee et al. demonstrated that both physicians and patients had positive experiences using an ePRO phone app to daily monitor RA disease activity [20]. Furthermore, another RCT by Li et al. demonstrated numerically improved disease control with another digital health application (Disease Activity Score 28 (DAS28)-CRP score difference between groups: 6.6%; 95% CI: 2.7%-10.4%; P = 0.001) [15].

Regarding improvement of pain, function, and work performance, some digital apps have shown promise. In an RCT by Rodríguez Sánchez-Laulhé et al., patients using the CareHand digital app for RA had improved hand function, work performance, and pain (P < 0.05) relative to patients not using the app [17]. Additionally, Haglo et al. found that self-administered app-based exercise programs improved high-intensity interval training outcomes in rheumatic disease patients to a similar degree relative to exertion under the direct guidance of healthcare professionals [16]. Regarding the safety and efficacy of RA smartphone applications, Seppen et al. found that patients randomized to an RA smartphone app for disease self-monitoring had non-inferior changes in DAS28-ESR scores relative to usual care and a 38% decrease reduction in consultations with a rheumatologist [19].

Further evidence suggests that asynchronous mobile health tools such as digital apps may provide a net benefit to patients. One systematic literature review by Seppen et al. explored the impact of various asynchronous mobile health tools (SMS reminders, website apps, smartphone apps, and pedometers) on several RA patient outcomes (medication compliance, achieving rapid remission, and more) [24]. Nearly all of the included studies demonstrated desirable outcomes; however, all studies demonstrated risk of bias in at least one Cochrane risk-of-bias domain.

Of course, the overall benefit of smartphone apps depends in large part on patient adherence. It is encouraging to note that, in a randomized trial by Colls et al., amongst 78 participants, there was high adherence to daily electronic patient-reported outcome (ePRO) questions (median: 79% (48-90% IQR)) [18]. However, factors such as patients being at least 65 years old (P = 0.03) and good disease control (low baseline Clinical Disease Activity Index (CDAI), P = 0.02) were strongly correlated with daily ePRO adherence. This suggests that younger patients with worse disease activity may not engage as well with smartphone apps.

Benefits of Digital Health in RA

Patient empowerment and engagement: Users appreciate having a better overview and understanding of their own disease activity. Electronic data collection from digital apps allows patient-rheumatologist interactions to strengthen and hence contribute to shared decision-making, as well as awareness of disease fluctuations [25]. RA patients perceive using specific digital apps to be supportive of their care, by providing users the potential for time-saving and paper reduction when recording their PRO.

Cost efficiency and resource optimization: Integrating telemedicine and remote monitoring via digital health for patients with RA brings significant cost-efficiency benefits, such as the reduction in need for frequent in-person hospital visits, which is particularly important in rheumatology, as many patients are immunosuppressed or suffer physical limitations inhibiting their ability to travel to appointments. Patients have the potential to receive consultations in the comfort of their homes, reducing the need for extensive travel and associated costs, especially for patients who live in rural towns. Reduction in financial burden would subsequently improve their overall quality of life.

Using digital health, data analytics obtained could help healthcare institutions allocate resources more efficiently by gaining insights into patient needs, disease progression, and treatment outcomes [26]. A study by Prinja et al. showed that there was a cost saving of USD 425 million in the use of the ReMiND program, a digital app focusing on reducing maternal and neonatal deaths, in which there was increased uptake of preventative services and better contact with the public health system [27]. Translating this to rheumatology and in RA patients, this will allow these institutions to allocate resources such as healthcare personnel and medications, especially biologics, in a timely manner.

Challenges and Ethical Considerations

Privacy in the digital age: With the advancement of digital technology, the availability of patient data on devices raises health information privacy concerns. In a systematic review by Luo et al., it was found that there were no official requirements to ensure that secure transmission of patient health data occurred during app development [22]. In this study, one observation was that, in general, there were limited measures that protected patient privacy (e.g., passwords), while there were many disclaimers in place that protected app developers from litigation. This raises concerns, given that private organizations usually do not offer and may not fully understand the level of privacy protections required by medical institutions. Technological development is rapidly progressing and could outpace the demands of safeguarding patient information and data [28].

Heterogeneous quality of digital applications: A systematic review performed by Najm et al. demonstrated various qualities of different digital applications, causing a discrepancy in assessment and results [29]. This may be due to poor app development processes where there is a lack of involvement of patients and healthcare providers in the creation of many of these apps [29]. It has been shown that healthcare providers are only involved in 35% (n=7) of the digital apps for patients with RA [22]. The involvement of healthcare professionals in the development of these apps is key to ensuring that accurate medical information can be communicated to patients remotely.

The quality of a digital app may alter how favourably it is perceived by patients. In a qualitative study by Knudsen et al., participants from an existing RCT were interviewed to determine the perceived impacts of an e-learning program. While participants noted a lack of relational support, individuals overall had positive experiences with e-learning for the purpose of better understanding their condition [30]. In contrast, a systematic review by Dantas et al. of mobile health applications in Brazil for rheumatic diseases found that many apps had limited credibility, levels of engagement, and user experiences, based upon the Mobile App Rating Scale (MARS) [21]. Moreover, a systematic review by Luo et al. found that most of the applications in their study did not provide a comprehensive patient experience [22]. This supports the notion that digital apps should undergo a centralized screening assessment to ensure quality control [30].

Additionally, it seems that altering specific app features may significantly increase app quality and patient experiences. For instance, in a systematic quality appraisal, Bearne et al. found a high-quality app called RAISE based on MARS that involved several behaviour change techniques and good aesthetics, which helped patients engage with physical activity and provided evidence-based information to patients with RA [23].

Some of these apps may also deliver varying quality of information to rheumatologists. For example, a study by Stenzel et al. involved RA patients performing a joint self-examination and reporting outcomes via a digital app. The quality of these joint self-examinations may differ significantly between patients, influencing clinical decision-making [31]. Therefore, there is a significant need for a standardized process in the development of these apps.

Discussion

It is evident that, although a wide range of mobile applications has been developed to support the management of RA, their rapid proliferation in the absence of standardized quality control measures poses challenges for clinical implementation. Among the six RCTs included in this review, the apps investigated demonstrated considerable variation in scope and functionality. One study evaluated an app designed primarily to deliver structured exercise programs, four studies assessed apps that monitored disease activity through daily patient-reported questionnaires and integration of laboratory results, and one trial examined a multifaceted app that combined exercise and education modules with disease activity monitoring. The outcomes of these RCTs were mixed: three demonstrated significant improvements in patient outcomes, while the remaining three reported no measurable differences between intervention and control groups. The four systematic reviews similarly identified a diverse collection of apps, with substantial heterogeneity in features, usability, and reported benefits. Notably, one review conducted in Brazil highlighted issues with suboptimal user interface and aesthetics, underscoring the importance of patient engagement and app design in driving adoption. Collectively, these findings highlight that, while digital health tools hold considerable promise in RA management, current evidence is fragmented due to inconsistencies in study design, outcome measures, and reporting. Future research should prioritize standardized, methodologically robust evaluations of digital health interventions, with particular attention to usability, patient-centered outcomes, and long-term integration into routine clinical care.

Limitations

As this was a scoping review, a formal risk of bias assessment was not conducted, consistent with the methodological framework by Arksey and O’Malley. Furthermore, data were only extracted from English databases and only if they were freely available. The goal was to map the existing literature rather than appraise study quality. However, we documented key methodological features of each study to provide context for the evidence base. A possible second systematic review would provide great benefit in extending this preliminary review that we have presented. Given the extreme pace of development of apps and technology, different apps provide different benefits, and some may not yet be studied or published. While these apps may someday be included in clinical practice, subsequent investigations and research of these apps are required before evidence-based recommendations can be made.

## Conclusions

The integration of digital applications holds immense promise for rheumatology, offering the potential to revolutionize assessment and management in RA. Digital health not only offers exciting ways to manage huge amounts of data but may also provide empowerment to patients to manage their own healthcare. Having said that, our principal findings suggest that most digital health applications in RA are heterogeneous in their features, with variable benefits based on their primary outcomes. Digital applications also come with possible risks, including the lack of protection of patient data. Additionally, the rate of technological development is currently outpacing the speed of regulation. In light of these findings, future studies should further assess whether the benefits of digital apps outweigh the potential drawbacks and should seek to assess the quality of app development with tools such as MARS. This will form the foundational steps of integrating digital health into our clinical practice for RA and in other rheumatological diseases.
